# Identification of *protease m1 zinc metalloprotease* conferring resistance to deltamethrin by characterization of an AFLP marker in *Culex pipiens pallens*

**DOI:** 10.1186/s13071-016-1450-4

**Published:** 2016-03-23

**Authors:** FF Zou, Q Guo, Y Sun, D Zhou, MX Hu, HX Hu, BQ Liu, MM Tian, XM Liu, XX Li, L Ma, B Shen, CL Zhu

**Affiliations:** Department of Pathogen Biology, Nanjing Medical University, 140 Hanzhong Road, Nanjing, Jiangsu 210029 PR of China

**Keywords:** *Protease m1 zinc metalloprotease*, Deltamethrin resistance, *Culex pipiens pallens*, AFLP, *CYP6CP1*

## Abstract

**Background:**

Continuous and excessive application of deltamethrin (DM) has resulted in the rapid development of insecticide resistance in *Culex pipiens pallens*. The quantitative trait loci (QTL) responsible for resistance to DM had previously been detected in *Cx. pipiens pallens*. But locating the QTLs on the chromosomes remained difficult. An available approach is to first characterize DNA molecular markers linked with the phenotype, and then identify candidate genes.

**Methods:**

In this study, the amplified fragment length polymorphism (AFLP) marker L3A8.177 associated with the QTL, was characterized. We searched for potential candidate genes in the flank region of L3A8.177 in the genome sequence of the closely related *Cx. pipiens quinquefasciatus* and conducted mRNA expression analysis of the candidate gene *via* quantitative real-time PCR. Then the relationship between DM resistance and the candidate gene was identified using RNAi and American CDC Bottle Bioassay in vivo. We also cloned the ORF sequences of the candidate gene from both susceptible and resistant mosquitoes.

**Results:**

The genes *CYP6CP1* and *protease m1 zinc metalloprotease* were in the flank region of L3A8.177 and had significantly different expression levels between susceptible and resistant strains. *Protease m1 zinc metalloprotease* was significantly up-regulated in the susceptible strains compared with the resistant and remained over-expressed in the susceptible field-collected strains. For deduced amino acid sequences of *protease m1 zinc metalloprotease*, there was no difference between susceptible and resistant mosquitoes. Knockdown of *protease m1 zinc metalloprotease* not only decreased the sensitivity of mosquitoes to DM in the susceptible strain but also increased the expression of *CYP6CP1*, suggesting the role of *protease m1 zinc metalloprotease* in resistance may be involved in the regulation of the P450 gene expression.

**Conclusion:**

Our study represents an example of candidate genes derived from the AFLP marker associated with the QTL and provides the first evidence that *protease m1 zinc metalloprotease* may play a role in the regulation of DM resistance in *Cx. pipiens pallens*.

**Electronic supplementary material:**

The online version of this article (doi:10.1186/s13071-016-1450-4) contains supplementary material, which is available to authorized users.

## Background

Vector-borne diseases account for over 17 % of all infectious diseases, causing more than 1 million deaths annually [1]. Mosquitoes, the best known disease vectors, transmit diseases including malaria, dengue, yellow fever, West Nile fever and filariasis [2]. Currently, there are no commercially available vaccines against malaria, West Nile fever and filariasis so vector control is considered important for the control of diseases, and the core strategies of it mainly rely on insecticides [3]. There were four classes of insecticides recommended by the WHO, namely organochlorines, organophosphates, carbamates and pyrethroids [4], and only pyrethroids were allowed for use on long-lasting insecticidal nets (LLINs) [5]. Deltamethrin (DM), an important synthetic pyrethroid insecticide, has been accorded wider acceptance during the past few years due to its high activity against insects, environmental safety and high vulnerability to enzymatic degradation [6]. Unfortunately, many countries rely heavily on the use of insecticides in controlling mosquito vectors. As a result, vector control campaigns in some areas are facing serious problems with the rise of insecticide resistance, which has become a major obstacle for mosquito control. Effective insecticide resistance management (IRM) is essential, and determination of the mechanisms underpinning insecticide resistance will greatly assist the development of much-needed novel strategies for IRM.

Resistance to pyrethroids is a complex polygenic phenotype and the two best understood mechanisms of insecticide resistance in mosquitoes are target site insensitivity and metabolic resistance [7–9]. A number of genes associated with insecticide resistance were reported, including cytochrome P450s, esterases, GST, sodium channel gene, G-protein-coupled receptor-related genes and serine proteases [10–12]. Indeed, none of the currently known genes can entirely explain the molecular basis for pyrethroid resistance. Moreover, insecticide exposure is a potent selective force and insecticide resistance is evolving. Identifying novel genes associated with pyrethroid resistance is critical for the effective control of mosquitoes.

A preliminary study using F_2_ progeny from crossing between a susceptible and a resistant strain of *Cx. pipiens pallens* identified seven deltamethrin-related QTLs. 12 AFLP markers flanking QTLs, in linkage with the resistance gene were cloned and blasted to a unique genomic position of the *Cx. pipiens quinquefasciatus* reference genome (supercontigs) [13]. *Cx. pipiens quinquefasciatus* and *Cx. pipiens pallens* are the two most common subspecies in the *Cx. pipiens* complex. Introgression between these species is common in the United States and the taxonomic status of this complex has been a subject of debate [14, 15]. The *Cx. pipiens quinquefasciatus* genome sequence was recently determined using the whole genome shotgun approach, thus providing a valuable resource for advancing genome studies in this species complex [16]. As the genome assembly of *Cx. pipiens quinquefasciatus* is represented by a high number of supercontigs with no order or orientation on the chromosomes. Locating the QTLs on the chromosomes remained difficult. An available approach is sequencing molecular markers associated with the QTL, then isolating and characterizing flanking genomic region [17]. In the present study, the genomic sequence surrounding the sequenced AFLP markers were used to search for candidate genes potentially related to insecticide resistance. Marker L3A8.177 in the vicinity of DR-4 (the QTL contributing 15 % to phenotypic variation) matched a non-coding region in the supercontig 3.388. Two candidate genes were found around the genomic sequence surrounding AFLP markers, *CYP6CP1* (VectorBase ID CPIJ012484; 119,517 bp away from the L3A8.177) and *protease m1 zinc metalloprotease* (VectorBase ID CPIJ012471; 386,339 bp from the L3A8.177), which were significantly differently expressed between susceptible and resistant strains of *Cx. pipiens pallens*. The *CYP6CP1* belongs to a member of CYP6 family, and there are many reports demonstrating the relationship between pyrethroid resistance and elevated activity of CYP6 family members in different mosquito species [18]. The *protease m1 zinc metalloprotease* belongs to families of zinc metalloproteases, involved in a variety of physiologically important processes such as protein processing and turnover, regulation of peptide hormone action, viral infection, tissue invasion, and cell cycle control [19, 20]. Moreover, the relationship between metalloproteases and biological insecticide resistance had been reported. Paris et al. identified a gene coding for an aminopeptidase potentially involved in resistance to *Bacillus thuringiensis israelensis* (Bti) toxins in mosquitoes by combining AFLP genome scans and 454 pyrosequencing [21]. And Lee et al. reported that aminopeptidases were altered in an insecticide resistant strain of *Aedes aegypti* using transcriptome sequencing [22]. As the association of *protease m1 zinc metalloprotease* with deltamethrin resistance in mosquito had not been reported, we focused on the study of *protease m1 zinc metalloprotease*.

We characterized *protease m1 zinc metalloprotease* in DM-resistant strains and susceptible strains in both laboratory and field populations. The expression profiles of *protease m1 zinc metalloprotease* in different mosquito life stages was also established. RNA interference strategy was used in susceptible mosquitoes to investigate the relationship between *protease m1 zinc metalloprotease* and DM resistance. The *protease m1 zinc metalloprotease* knockdown not only decreased the sensitivity of mosquito to DM but also increased the expression of *CYP6CP1*, suggesting that *protease m1 zinc metalloprotease* may regulate mosquito DM resistance through modulating detoxification metabolism.

## Methods

### Mosquito strains

Several laboratory-reared strains of *Cx. pipiens pallens* were used in this study: a deltamethrin-susceptible (Lab-DS) strain (the 50 % lethal concentration LC_50_ = 0.04 mg/L) and 4 deltamethrin-resistant (Lab-DR) strains of different resistance levels (Lab-DR1, Lab-DR2, Lab-DR3, Lab-DR4). The LC_50_ of 4 Lab-DR strains was 0.31 mg/L, 0.85 mg/L, 3.03 mg/L, 3.43 mg/L, respectively. The Lab-DS strain was collected from Tangkou town of Shandong Province (35.12 N; 116.50 E) in 2009 and reared without exposure to any insecticide. The Lab-DR strains were selected from the Lab-DS strain, and the selection detail was showed previously [13, 23]. In addition, three samples were made of various structures in female adults (leg, head, thorax, abdomen). The individuals were obtained over all life stages from both Lab-DS and Lab-DR4 strains. We also collected five field populations of *Cx. pipiens pallens* from Shangdong Province: PY (Pingying City, 36.29 N; 116.42E), SH (Shanghe City, 37.31 N; 117.15E), GD (Gudao City, 37.85 N; 118.81E), HM (Huiming City, 37.45 N; 117.41E), JN (Jining City, 35.26 N; 116.35E). For each field mosquito population, larvae were brought back to the insectary after morphological identification. To distinguish susceptible and resistant strains of field populations, female adults were exposed to discriminating doses of deltamethrin (0.05 %) for susceptibility tests following the standard WHO testing protocol [24]. The mosquitoes that survived the 24-h recovery period were classified as DM resistant, while those knocked down in an hour during the bioassay were classified as DM susceptible. All mosquito samples were preserved for further analysis at −80 °C.

### RNA extraction, cDNA synthesis and quantitative real-time PCR analyses

Total RNA was extracted from all mosquitoes (Lab-DS, Lad-DR1, Lab-DR2, Lab-DR3 and Lab-DR4; susceptible and resistant ones of five field strains). The method of RNA extraction and cDNA synthesis was as the description of Zhou et al. [25].

All quantitative real-time PCR (qRT-PCR) analysis mentioned in the article was as the following description. The experimental technique was performed on the ABI PRISM 7300 (Applied Biosystems, USA) using Power SYBR Green PCR Master Mix (Applied Biosystems, USA) according to the manufacturer’s protocol. The qRT-PCR primers were designed using Primer 5.0 (Additional file 1), based on the corresponding cDNA sequences obtained from the VectorBase. The qRT-PCR analysis was performed using the method described previously [26]. The PCR products were used for melting curve and agarose gel electrophoresis analysis to confirm their amplification specificity. The assays were conducted in triplicate, and the average value of the triplicate was used for analysis of gene expression differences. The *β-actin* and *RsP7* genes were used as the standard for expression normalization for each gene because of their stable expression [27, 28].

### RNAi analysis and American CDC bottle bioassay

Double-stranded RNA (dsRNA) for two genes and negative control (NC) RNA were designed and synthesized from GenePharma (GenePharma, Shanghai, China) at a concentration of 20 μM (The sequences were in Additional file 2, siRNA404 for *CYP6CP1*, siRNA345 for *protease m1 zinc metalloprotease*). Approximately 350 ng of dsRNA or NC RNA was injected into 1-day-old female mosquitoes (siRNA404 into the Lab-DR4 strain, siRNA345 into the Lab-DS strain). The procedure of microinjection on RNAi assays were carried out according to standard methodology [29, 30]. For one biological replicate, 30 female adults were injected with dsRNA, 30 were injected with NC RNA, and another 30 were non-injected. Three biological replicates were performed. The groups were kept in the insectary at 28–30 °C in a 16-h light/8-h dark photoperiod with 70–80 % humidity for 3 days. Then we verified the RNAi efficiency using qRT-PCR and detected the relative mortality of adult female mosquitoes under DM exposure by American CDC bottle bioassay. We conducted the CDC bottle bioassay as a guideline for evaluating insecticide resistance in vectors. (http://www.cdc.gov/parasites/education_training/lab/bottlebioassay.html). The diagnostic doses of DM applied in this study were 4 mg for Lab-DR4 and 0.01 mg for Lab-DS, per bottle (250 mL) of 20–25 individuals. The control bottle coated with acetone was with dsRNA-microinjected individuals. The number of dead mosquitoes was recorded every 15 min, up to 2 h. At last the mortality rate was calculated at the diagnostic time.

### Cloning and sequencing for *protease m1 zinc metalloprotease*

The open reading frame (ORF) of *protease m1 zinc metalloprotease* was amplified using a pair of specific primers: forward primer: 5‘- ATGAACAACAGCAACAACGCCAAGA-3’ and reverse primer: 5‘- CTACGGGTACTTTTCTGGCATAGTT-3’, which were designed based on the cDNA sequence of *Cx. pipiens quinquefasciatus* (https://www.vectorbase.org/Culex_quinquefasciatus/Transcript/Sequence_cDNA?db=core;g=CPIJ012471). PCR was performed using PrimeSTAR® HS DNA Polymerase (Code No. R010A, TaKaRa, Japan) following the manufacturer’s recommendations in both Lab-DS and Lab-DR4 strains. Amplified products were electrophoresed on a 1 % agarose gel, and target bands were excised and purified using TaKaRa MiniBEST Agarose Gel DNA Extraction Kit Ver. 4.0 (Code No. 9762, TaKaRa). Purified product was cloned into the pMD-19 T Simple Vector. Recombinant plasmid DNA was isolated and sequenced by the Beijing Genomic Institute. Sequences were accepted if identical from three different individuals from one strain.

### Statistical analysis

The statistical significance of the gene expression was calculated using a Student’s t-test [31] and mosquito mortality was analyzed using the chi-square test [32], by the Social Sciences (SPSS) software. Linear regression analysis was used to correlate transcription level and DM resistance level (LC_50_) in *Cx. pipiens pallens* [25, 31]. A value of *P* < 0.05 was considered statistically significant.

## Results and discussion

### Screening of candidate genes in the flank of the marker

The AFLP marker L3A8.177 matched a non-coding region in the supercontig 3.388 (3.388:393,117–393,273; Score = 308, Except = 9e-74, Identity = 99.37 %) [13]. There were 18 genes in the flank region of the marker in supercontig 3.388 (Additional file 3). By combination the molecular functions and biological processes of genes (https://www.vectorbase.org/search) with a keyword search (insecticide resistance) in the NCBI database (http://www.ncbi.nlm.nih.gov/pubmed/), we selected five genes (CPIJ012485, CPIJ012484, CPIJ012475, CPIJ012472 and CPIJ012471) as potential candidate genes.

**Fig. 1:**
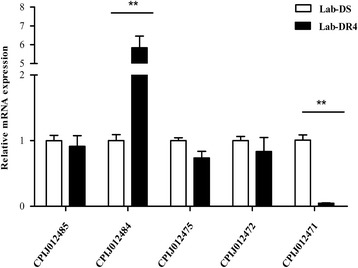
Screening potential candidate genes *via* quantitative real-time PCR in *Culex pipiens pallens*. The relative expression levels of five genes were detected in adult female mosquitoes of the Lab-DS (LC_50_ = 0.04 mg/L) and Lab-DR4 (LC_50_ = 3.43 mg/L) strains. The gene CPIJ012484 (*CYP6CP1*) was significantly over-expressed in the resistant strain while gene CPIJ012471 (*protease m1 zinc metalloprotease*) was under-expressed. The results were shown as the mean ± S.E. **P < 0.01 compared with the Lab-DS strain

The transcription profiles of five genes associated with the marker were examined using qRT-PCR. As shown in Fig. 1, the Lab-DR4 strain (LC_50_ = 3.43 mg/L) had a higher expression level for CPIJ012484 (*CYP6CP1*) (*t*_(4)_ = 4.34, *P* = 0.006), while a lower expression level for CPIJ012471 (*protease m1 zinc metalloprotease*) (*t*_(4)_ = 51.23, *P* < 0.0001), compared to the Lab-DS strain. Moreover, this result matched the report by Lv et al., that the range of expression level between the resistant and susceptible strain was a 1.3-fold difference for *CYP6CP1*, and 0.75-fold for *protease m1 zinc metalloprotease* using comparative transcriptome analyses by RNA sequence in *Cx. pipiens pallens* [10].

In recent years, the availability of tightly linked robust PCR-based markers has been used extensively to map and tag candidate genes of quantitative traits in many species. For example, Lyons et al. characterized AFLP markers associated with growth in *Marsupenaeus japonicus*, and identified a candidate gene surrounding the AFLP band 7.21a [17]. And Podio et al. obtained the flanking regions of a group of molecular markers linked to apospory, in order to identify possible candidate genes in *Paspalum notatum* [33]. In a previous report, we obtained genomic positions of several AFLP markers linked to QTLs referring DM resistance in the *Cx. pipiens quinquefasciatus* reference genome. And here we obtained two genes in the flank region of the marker L3A8.177, showing the different expression levels among Lab-DS and Lab-DR4 strains. Our study demonstrated AFLP marker is a relatively efficient marker for the genetic architecture analysis of quantitative traits.

Our finding detected that *CYP6CP1* had a significantly higher expression in the resistant strain compared with the susceptible strain. To date, increased P450-mediated detoxification has been found in many insect species associated with enhanced metabolic detoxification of insecticides, by the increased levels of P450 proteins and P450 activity that result from constitutively transcriptional over expression of P450 genes in insecticide resistant insects [9, 34–37]. And the members of CYP6 family have been verified to be involved in pyrethroid metabolism [23, 38–40]. The result of *CYP6CP1* was in keeping with the verification. However, *protease m1 zinc metalloprotease* was only reported to be differently expressed between susceptible and resistant mosquitoes by the transcriptom studies, and we chose the gene for further study.

### In vivo validation by RNAi and American CDC bottle bioassay

**Fig. 2:**
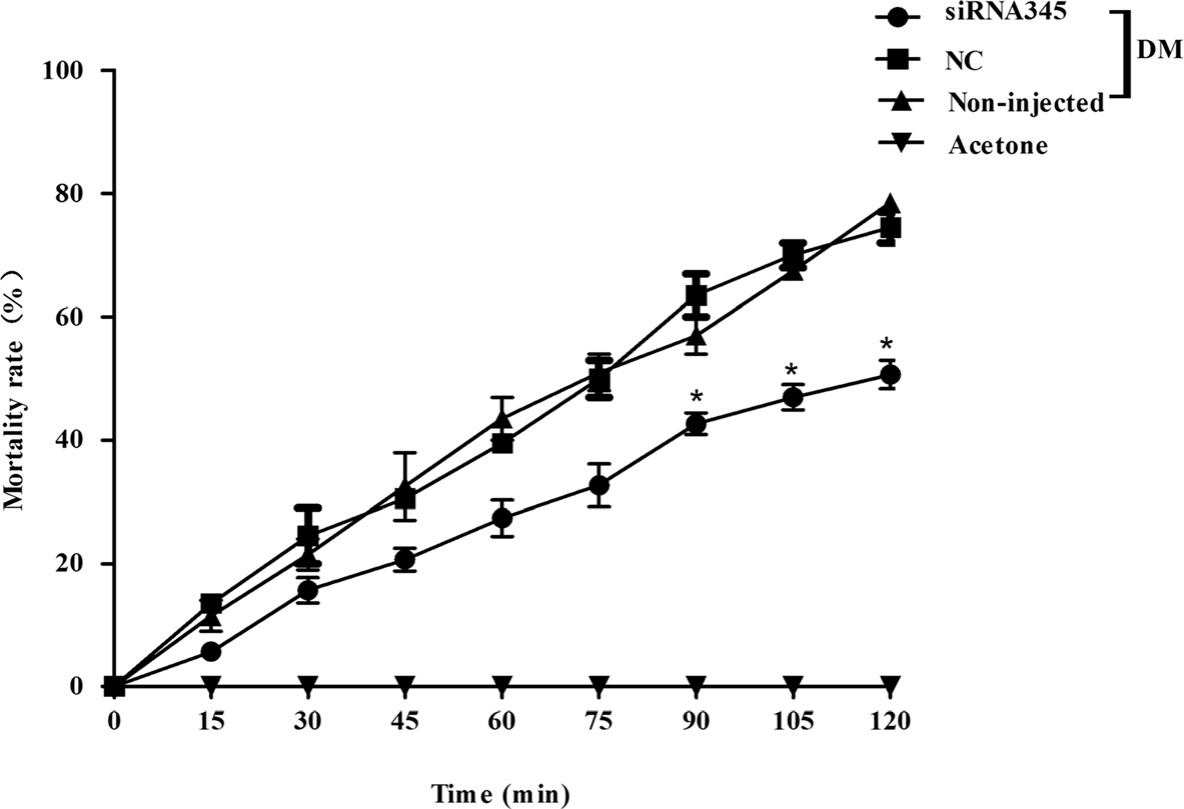
Functional study of *protease m1 zinc metalloprotease* in the Lab-DS strain. Mortalities of microinjected mosquitoes were observed after a 2 h exposure to American CDC bottles treated with deltamethrin (0.01 mg/ml). The siRNA345 microinjected group had a relatively lower mortality rate than the negative control (NC) and non-injected groups. The figures show the mean ± SD of three independent experiments (*P < 0.05)

To evaluate the involvement of *protease m1 zinc metalloprotease* in DM resistance in vivo, we sought to specifically inhibit the expression of the gene by injecting its corresponding dsRNA into the thorax of the adult female mosquitoes. The expression of *protease m1 zinc metalloprotease* decreased 50 % by siRNA345, compared with that in the corresponding NC group, separately (*t*_(4)_ = 3.19, *P* = 0.016) (Additional file 4). The consequence of decrease in gene transcript on the susceptibility of female adults against DM was examined by the American CDC Bottle Bioassay (Fig. 2). For *protease m1 zinc metalloprotease* in the Lab-DS strain, the siRNA345 group had a lower mortality rate compared to the other groups. And knockdown of the gene decreased the sensitivity of mosquitoes to DM.

### Cloning the ORF sequence of *protease m1 zinc metalloprotease*

We cloned the ORF regions of *protease m1 zinc metalloprotease* in Lab-DS and Lab-DR4 strains. The ORF region has 924 bp and encodes for a protein with 308 amino acids (Additional file 5). The *protease m1 zinc metalloprotease* of *Cx. pipiens pallens* shared the highest homology with *Cx. pipiens quinquefasciatus* (94 % identity) by the standard protein/protein BLAST sequence comparison program (http://beta.uniprot.org/?tab=blast). An ORF sequence analysis carried out in two strains showed that only point mutation was found at the 806th base, and the mutation (806 T > C) was synonymous. The CDD software for structure analysis (http://www.ncbi.nlm.nih.gov/Structure/cdd/wrpsb.cgi) was used, demonstrating that the putative protein contains a domain: M1_APN_2 domain, from positions 150th to 291th (E-value: 2.16e-21).

The *protease m1 zinc metalloprotease* is a member of the peptidase family M. It preferentially cleaves neutral amino acids from the N-terminus of oligo peptides and is present in a variety of tissues and cell types [41]. And the family has been identified in many parasites and vectors. For example, zinc-metalloprotease GP63 of *Leishmania*, a critical virulence factor, has been suggested to modulate cellular signaling through the subversion of host protein tyrosine phosphatase (PTP) function [42], significantly inhibits NLRP3 inflammasome function and IL-1β production [43]. In insects, aminopeptidase Ns (APNs) were also putative Cry toxin receptors (Cry proteins are pore-forming toxins that bind to the midgut epithelial cell membrane of susceptible insect larvae, causing extensive damage) [44–46]. In our study, the *protease m1 zinc metalloprotease* gene of the Lab-DS strain exhibited a significantly higher mRNA level than the highly resistant strain. The injection of dsRNA corresponding to the gene sequence resulted in a significant gene knockdown effect in the susceptible strain, as evidenced by the presence of a significantly lower susceptibility to DM than in the control groups. But for deduced amino acid sequences of *protease m1 zinc metalloprotease*, there was no difference between susceptible and resistant mosquitoes. The understanding of how resistance evolves at the molecular level is known predominantly to be involved in amplification, over-expression, and coding sequence variation of genes related to mechanisms of insecticide resistance [18]. These data strongly suggested that the role of *protease m1 zinc metalloprotease* in *Cx. pipiens pallens* resistance to DM may be by the change of the transcription level.

### Expression level analysis for *protease m1 zinc metalloprotease*

**Fig. 3:**
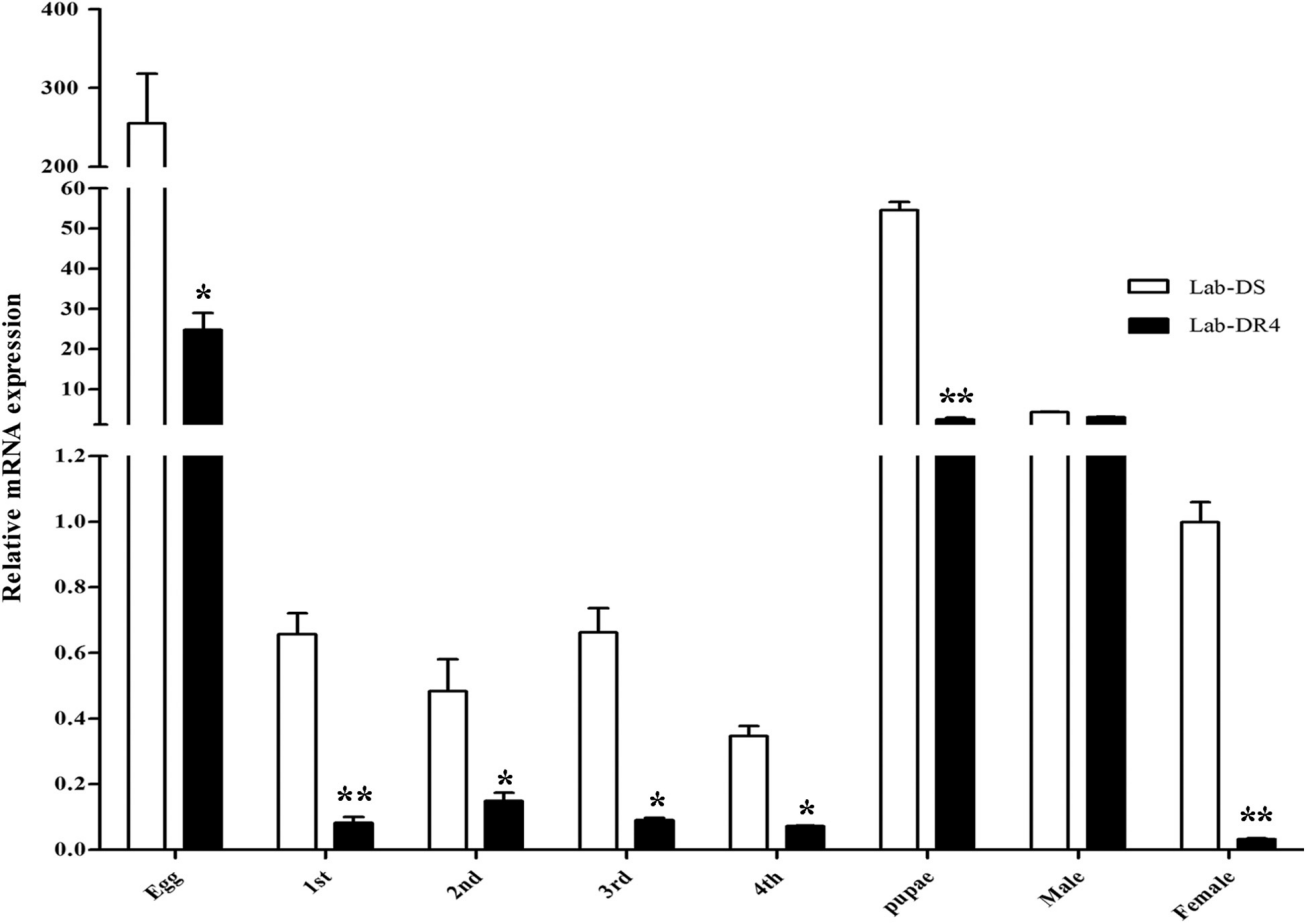
Expression levels of *protease m1 zinc metalloprotease* at all development stages. The gene expression level at the female stage in Lab-DS strain was considered to be 1. The figures show the mean ± SD of three independent experiments. *P < 0.05, **P < 0.01 compared with the Lab-DS strain

Over the life cycle of the mosquitoes, the relative RNA expression levels at all developmental stages (egg, L1-L4 instar larvae, pupae, male and female) of *Cx. pipiens pallens* were given in Fig. 3. The gene was transcribed at all stages but most in egg. And the expression levels were relatively low in larvae. It was worth mentioning that *protease m1 zinc metalloprotease* was significantly up-regulated at different stages (egg: *t*_(4)_ = 3.66, *P* = 0.029; L1: *t*_(4)_ = 6.96, *P* = 0.002; L2: *t*_(4)_ = 3.29, *P* = 0.030; L3: *t*_(4)_ = 6.81, *P* = 0.019; L4: *t*_(4)_ = 3.02, *P* = 0.039; pupae: *t*_(4)_ = 5.41, *P* = 0.006; female: *t*_(4)_ = 51.23, *P* < 0.0001), except for the male adult, in the Lab-DS strain compared with the Lab-DR4 strain.

**Fig. 4:**
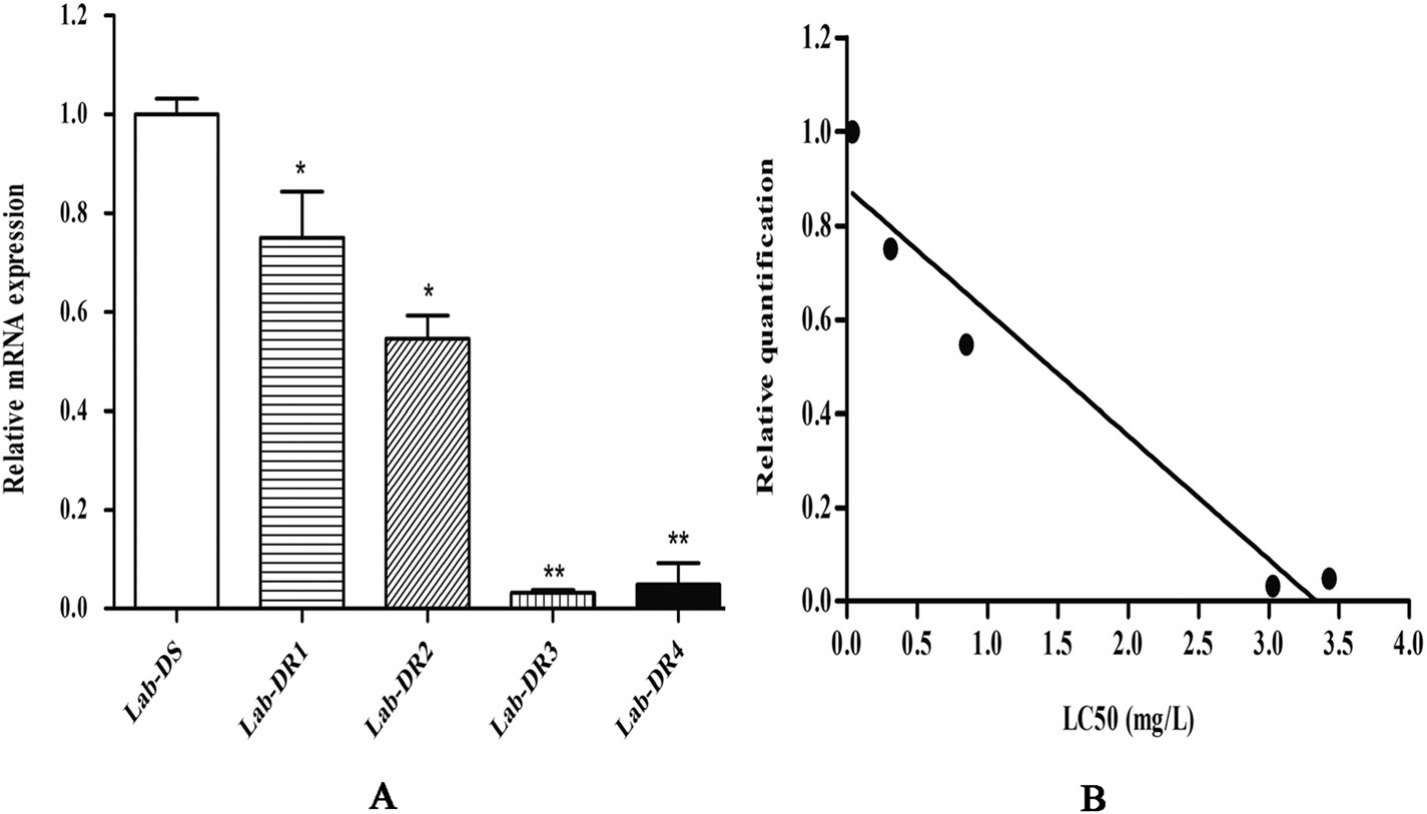
**a** Expression profiles of *protease m1 zinc metalloprotease* transcripts in five strains with different deltamethrin resistance levels. The results were shown as the mean ± S.E. Significant differences indicated by *(*P* < 0.05) and **(*P* < 0.01) were compared with the Lab-DS strain. **b** The relationship between *protease m1 zinc metalloprotease* transcriptional levels and deltamethrin resistance of *Culex pipiens pallens*. The Y-axis was the ratio of the gene expression in Lab-DR mosquitoes compared with that in Lab-DS mosquitoes, *r* = −0.97, *P* < 0.05

The expression levels of *protease m1 zinc metalloprotease*　in five strains with different levels of DM resistance were shown in Fig. 4. With the increase of resistance levels in lab strains (DS = 0.04, DR1 = 0.31, DR2 = 0.85, DR3 = 3.03, DR4 = 3.43), transcriptional expressions of the gene gradually decreased (Fig. 4a). When the LC_50_ was increased 75-fold, the transcriptional expression was decreased by approximately 97 % (*t*_(4)_ = 50.53, *P* < 0.0001). We used the correlation analysis and found a significant negative correlation between relative quantification of *protease m1 zinc metalloprotease* (*Y*) and the LC_50_ (*X*) (*r*_(xy)_ = −0.974, *P* = 0.005, Fig. 4b). The correlation coefficient indicated that the gene may play a role in the development of deltamethrin resistance in *Cx. pipiens pallens*.

**Fig. 5:**
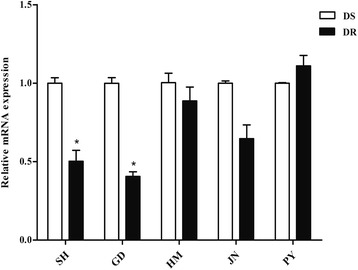
The expression analysis of *protease m1 zinc metalloprotease* in field-collected strains of *Culex pipiens pallens*. The results were shown as the mean ± S.E. Significant differences indicated by *(*P* < 0.05) were compared with the corresponding susceptible strain

We then expanded mosquito samples to identify the expression level in field-collected strains. The *protease m1 zinc metalloprotease* transcriptional expression was analyzed in SH, GD, HM, JN, PY field-collected populations, separately (Fig. 5). In PY population, the comparison between susceptible and resistant mosquitoes indicated no significant differences in transcription. But for the other populations, the gene was down-regulated in the resistant strain of the populations compared to the corresponding susceptible populations (0.50-fold in SH, 0.41-fold in GD, 0.88-fold in HM, 0.64-fold in JN, respectively), which was consistent with the result of the lab strains. The gene may be used as a potential genetic marker to monitor the pyrethroid resistance.

**Fig. 6:**
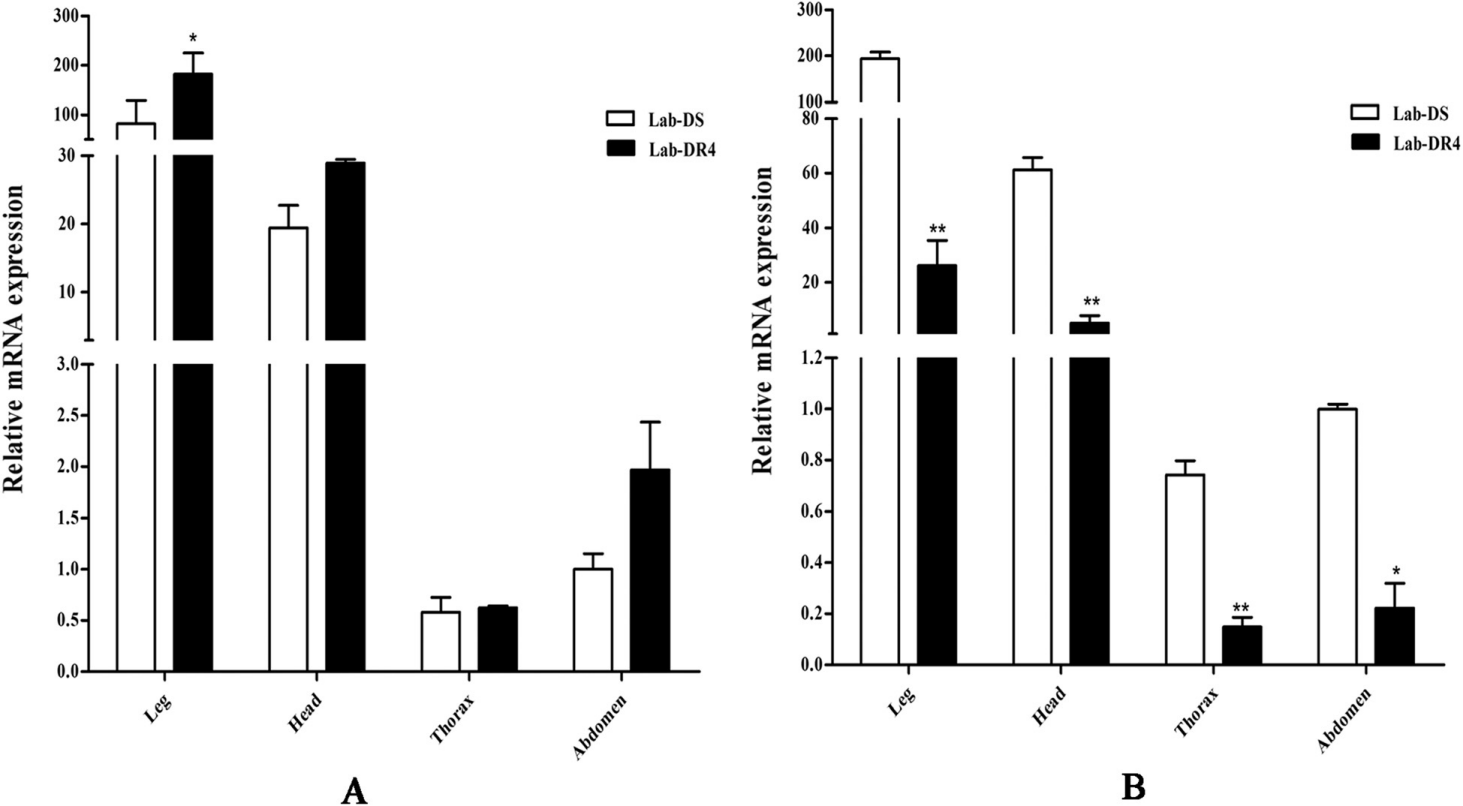
**a** Expression levels of *CYP6CP1* in various structures of *Culex pipiens pallens.* The gene expression level of the abdomen in Lab-DS strain was considered to be 1. The figures show the mean ± SD of three independent experiments. *P < 0.05, **P < 0.01 compared with the Lab-DS strain. **b** Expression levels of *protease m1 zinc metalloprotease* in various structures of *Culex pipiens pallens.* The gene expression level of the abdomen in Lab-DS strain was considered to be 1. The figures show the mean ± SD of three independent experiments. *P < 0.05, **P < 0.01 compared with the Lab-DS strain

We compared the expression of *protease m1 zinc metalloprotease* at different life stages (egg, larvae, pupae, adult males and females) between the Lab-DS and Lab-DR4 strains, in five lab strains with different DM-resistance levels, and in five different field-collected strains. The results showed that the gene maintained a high expression in the susceptible strain. Notably, the regression analysis performed to determine the relationship of transcriptional levels of *protease m1 zinc metalloprotease* and the LC_50_ of deltamethrin revealed a statistically significant negative relationship (*r*_(xy)_ = −0.974, *P* = 0.005). And it was reported that the midgut transcript levels of two APN genes (AAEL008158 and AAEL008162, belonging to the peptidase family M1), were significantly reduced in insecticide resistant *Aedes aegypti* larvae compared to the susceptible ones [22]. Aminopeptidases are widely distributed throughout the insect kingdom. In our lab strains of *Cx. pipiens pallens*, the gene expression of *protease m1 zinc metalloprotease* was in various structures but mainly in legs and head (Fig. 6b), which suggested the mechanism of this zinc metalloprotease involved in the DM resistance was different from the mechanism of APNs involved in Bti resistance [47, 48].

### The relationship of mRNA level analysis between *CYP6CP1* and *protease m1 zinc metalloprotease*

**Fig. 7:**
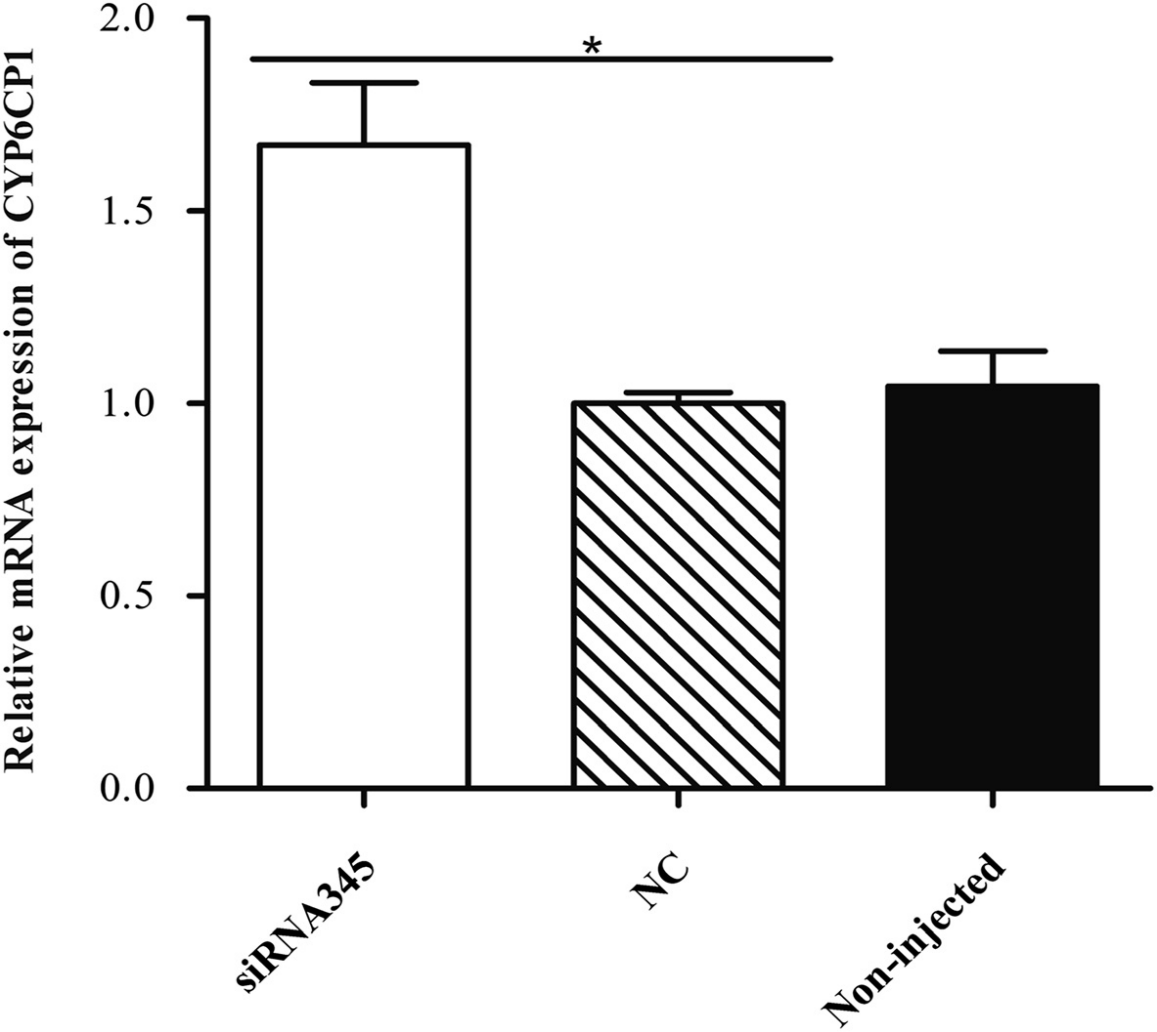
Relative expression of *CYP6CP1* in mosquitoes injected with dsRNA of *protease m1 zinc metalloprotease*. The relative gene expression of *CYP6CP1* along the Y-axis was the ratio of the gene expression in siRNA345-injected (dsRNA of *protease m1 zinc metalloprotease*) mosquitoes compared with that in NC-injected mosquitoes. The results were shown as the mean ± S.E. Significant differences were indicated by *(*P* < 0.05)

In our study, the expression level of *CYP6CP1* was up-regulated while the level of *protease m1 zinc metalloprotease* was down-regulated in the DM-resistance strain of *Cx. pipiens pallens* compared with the susceptible one. And the expression level of *CYP6CP1* was up-regulated when *protease m1 zinc metalloprotease* was knocked down in susceptible adults (*t*_(4)_ = 4.054, *P* = 0.015) (Fig. 7). We detected that knockdown of *CYP6CP1* increased the sensitivity of mosquitoes to DM (Additional file 6). Meanwhile, we analyzed the expression profile of *CYP6CP1* in various structures and detected the gene was mainly in legs and head, which was similar to the expression of *protease m1 zinc metalloprotease* (Fig. 6).

It has been proved that increased P450-mediated detoxification is a major mechanism of pyrethroid resistance in insects [49, 50], but the regulatory mechanisms of P450 up-regulation are still unclear. Metalloproteases play essential roles in regulation of metastasis, signal-transduction, spatial proteolytic activity and modulation of protein-protein and cell-cell interactions [51–53]. Mosha et al. showed that metalloprotease could be a regulatory factor for CYP2J2 in the hyperhomocysteinemia [54]. It was also reported that overexpression of CYP4A11 significantly induced invasion and expression of the MMP-9 (MMPs are classified as zinc-dependent proteinases) [55]. In our study, we detected that knockdown of *protease m1 zinc metalloprotease* increased the expression of *CYP6CP1*. So we speculated that *protease m1 zinc metalloprotease* may be involved in DM resistance by affecting the transcriptional level of *CYP6CP1*. Further experiments are needed to investigate the regulatory role of *protease m1 zinc metalloprotease*.

## Conclusions

In summary, *protease m1 zinc metalloprotease* was identified as a novel DM-resistance-associated gene using transcription analysis and RNAi, from the flanking sequences of the marker L3A8.177 in the genome sequence of the closely related *Cx. pipiens quinquefasciatus*. This study has shed new light on the potential function of *protease m1 zinc metalloprotease* in DM resistance and its regulatory function on resistance-related P450 gene expression. However, the entire regulatory pathway remains largely unclear. The work not only lays a foundation for identification of DM resistance genes in *Cx. pipiens pallens* by a genetic marker, but also identification of a novel deltamethrin-resistance-associated gene.

## Additional files

Additional file 1: List of the primers for qRT-PCR. (DOC 30 kb) Additional file 2: List of the siRNA sequences for RNAi. (DOC 27 kb) Additional file 3: Details of genes in supercontig 3.388 of the *Culex pipiens quinquefasciatus* genome. (DOC 44 kb) Additional file 4: RNAi efficiency verification of *protease m1 zinc metalloprotease* by qRT-PCR. The expression level of *protease m1 zinc metalloprotease* was down-regulated about 50 % in Lab-DS mosquitoes microinjected with the siRNA345, compared with that in mosquitoes injected NC RNA. The results were shown as the mean ± S.E. The significant difference was indicated by *(*P* < 0.05). (TIF 3435 kb) Additional file 5: The ORF sequences of *protease m1 zinc metalloprotease* in Lab-DS and Lab-DR4 strains. (DOCX 12 kb) Additional file 6: (**A**) RNAi efficiency verification of *CYP6CP1* by qRT-PCR. The expression level of *CYP6CP1* gene was down-regulated about 65 % in Lab-DR4 mosquitoes microinjected with the siRNA404, compared with that in mosquitoes injected NC RNA. (**B**) Functional study of *CYP6CP1* in Lab-DR4 strain. Mortalities of microinjected mosquitoes were observed after a 2 h exposure to CDC bottles treated with deltamethrin (4 mg/ml). The siRNA404 microinjected group had a higher mortality rate than the negative control. (TIF 1066 kb)
