# Supplementary material for: Identification of protease m1 zinc metalloprotease conferring resistance to deltamethrin by characterization of an AFLP marker in Culex pipiens pallens
Source: Parasit Vectors. 2016 Mar 23;9:172. doi: 10.1186/s13071-016-1450-4 (PMC4806500; doi:10.1186/s13071-016-1450-4)
Supplement: Additional file 1: — List of the primers for qRT-PCR. (DOC 30 kb) [file 13071_2016_1450_MOESM1_ESM.doc]

**Additional File 1**

**List of the primers for qRT-PCR**

| **Gene** | **Forward primer (5' to 3')** | **Reverse primer (5' to 3')** | **Length** |
| --- | --- | --- | --- |
| **CPIJ012471** | GCGACAACATAACACTTCACG | GACGGTTCGATTTCCTCCA | 174bp |
| **CPIJ012472** | GGAAGCTGTATCGAGGTGC | CGTCTGGTGATTGTCCATTAGT | 81bp |
| **CPIJ012475** | CGAGCTGCACAGCGATAAGA | CGGGAAGGACGGTGAAAGTA | 91bp |
| **CPIJ012484** | AAGCGGGAGATTGTGTTGA | CGGTTGATGTGGTTGTTGTT | 101bp |
| **CPIJ012485** | TGCTTGCTGACTTTTGGGT | AGTTGCGGGACTGTTTGTG | 168bp |
| ***β-actin*** | AGCGTGAACTGACGGCTCTG | ACTCGTCGTACTCCTGCTTGG | 153bp |
| ***RsP7*** | CCTGGAGCTGGAGATGAACT | ACGATGGCCTTCTTGTTGTT | 99bp |
