# Supplementary material for: Identification of protease m1 zinc metalloprotease conferring resistance to deltamethrin by characterization of an AFLP marker in Culex pipiens pallens
Source: Parasit Vectors. 2016 Mar 23;9:172. doi: 10.1186/s13071-016-1450-4 (PMC4806500; doi:10.1186/s13071-016-1450-4)
Supplement: Additional file 2: — List of the siRNA sequences for RNAi. (DOC 27 kb) [file 13071_2016_1450_MOESM2_ESM.doc]

**Additional File 2**

**List of the siRNA sequences for RNAi**

| **Name** | **Sense(5' to 3')** | **Antisense (5' to 3')** |
| --- | --- | --- |
| **NC** | GCGACGAUCUGCCUAAGAUdTdT | AUCUUAGGCAGAUCGUCGCdTdT |
| **siRNA345** | GGCCAAACUGGAUGAACAUTT | AUGUUCAUCCAGUUUGGCCTT |
| **siRNA404** | GGAAGAAGGCAGAGAGUAATT | UUACUCUCUGCCUUCUUCCTT |
