# Supplementary material for: Identification of protease m1 zinc metalloprotease conferring resistance to deltamethrin by characterization of an AFLP marker in Culex pipiens pallens
Source: Parasit Vectors. 2016 Mar 23;9:172. doi: 10.1186/s13071-016-1450-4 (PMC4806500; doi:10.1186/s13071-016-1450-4)
Supplement: Additional file 3: — Details of genes in supercontig 3.388 of the Culex pipiens quinquefasciatus genome. (DOC 44 kb) [file 13071_2016_1450_MOESM3_ESM.doc]

**Additional File 3**

**Details of genes in supercontig 3.388 of the *Culex pipiens quinquefasciatus* genome**

| **Gene No.** | **Description** | **Location** |
| --- | --- | --- |
| CPIJ012489 | conserved hypothetical protein | [SuperContig supercont3.388: 353,000-354,039](https://www.vectorbase.org/Culex_quinquefasciatus/Location/View?db=core;g=CPIJ012489;r=supercont3.388:353000-354039;t=CPIJ012489-RA) |
| CPIJ012488 | ribonuclease H1 | [SuperContig supercont3.388: 352,618-352,916](https://www.vectorbase.org/Culex_quinquefasciatus/Location/View?db=core;g=CPIJ012488;r=supercont3.388:352618-352916;t=CPIJ012488-RA) |
| CPIJ012487 | estrogen-related receptor | [SuperContig supercont3.388: 298,082-304,260](https://www.vectorbase.org/Culex_quinquefasciatus/Location/View?db=core;g=CPIJ012487;r=supercont3.388:298082-304260;t=CPIJ012487-RA) |
| CPIJ012486 | conserved hypothetical protein | [SuperContig supercont3.388: 274,005-289,413](https://www.vectorbase.org/Culex_quinquefasciatus/Location/View?db=core;g=CPIJ012486;r=supercont3.388:274005-289413;t=CPIJ012486-RA) |
| CPIJ012485 | NMD protein | [SuperContig supercont3.388: 273,645-273,936](https://www.vectorbase.org/Culex_quinquefasciatus/Location/View?db=core;g=CPIJ012485;r=supercont3.388:273645-273936;t=CPIJ012485-RA) |
| CPIJ012484 | *CYP6CP1* | [SuperContig supercont3.388: 270,865-273,600](https://www.vectorbase.org/Culex_quinquefasciatus/Location/View?db=core;g=CPIJ012484;r=supercont3.388:270865-273600;t=CPIJ012484-RA) |
| CPIJ012482 | conserved hypothetical protein | [SuperContig supercont3.388: 170,907-175,011](https://www.vectorbase.org/Culex_quinquefasciatus/Location/View?db=core;g=CPIJ012482;r=supercont3.388:170907-175011;t=CPIJ012482-RA) |
| CPIJ012481 | proacrosin, putative | [SuperContig supercont3.388: 167,691-170,768](https://www.vectorbase.org/Culex_quinquefasciatus/Location/View?db=core;g=CPIJ012481;r=supercont3.388:167691-170768;t=CPIJ012481-RA) |
| CPIJ012480 | synaptic vesicular amine transporter | [SuperContig supercont3.388: 162,596-164,056](https://www.vectorbase.org/Culex_quinquefasciatus/Location/View?db=core;g=CPIJ012480;r=supercont3.388:162596-164056;t=CPIJ012480-RA) |
| CPIJ012479 | conserved hypothetical protein | [SuperContig supercont3.388: 159,207-161,687](https://www.vectorbase.org/Culex_quinquefasciatus/Location/View?db=core;g=CPIJ012479;r=supercont3.388:159207-161687;t=CPIJ012479-RA) |
| CPIJ012478 | conserved hypothetical protein | [SuperContig supercont3.388: 149,154-151,175](https://www.vectorbase.org/Culex_quinquefasciatus/Location/View?db=core;g=CPIJ012478;r=supercont3.388:149154-151175;t=CPIJ012478-RA) |
| CPIJ012477 | cDNA sequence, putative | [SuperContig supercont3.388: 144,815-145,580](https://www.vectorbase.org/Culex_quinquefasciatus/Location/View?db=core;g=CPIJ012477;r=supercont3.388:144815-145580;t=CPIJ012477-RA) |
| CPIJ012476 | conserved hypothetical protein | [SuperContig supercont3.388: 143,352-144,259](https://www.vectorbase.org/Culex_quinquefasciatus/Location/View?db=core;g=CPIJ012476;r=supercont3.388:143352-144259;t=CPIJ012476-RA) |
| CPIJ012475 | ubiquitin-protein ligase | [SuperContig supercont3.388: 71,668-99,955](https://www.vectorbase.org/Culex_quinquefasciatus/Location/View?db=core;g=CPIJ012475;r=supercont3.388:71668-99955;t=CPIJ012475-RA) |
| CPIJ012474 | microtubule binding protein, putative | [SuperContig supercont3.388: 62,204-69,178](https://www.vectorbase.org/Culex_quinquefasciatus/Location/View?db=core;g=CPIJ012474;r=supercont3.388:62204-69178;t=CPIJ012474-RA) |
| CPIJ012473 | microtubule binding protein, putative | [SuperContig supercont3.388: 45,710-61,943](https://www.vectorbase.org/Culex_quinquefasciatus/Location/View?db=core;g=CPIJ012473;r=supercont3.388:45710-61943;t=CPIJ012473-RA) |
| CPIJ012472 | UDP-N-acetylglucosaminetransferase subunit alg13 | [SuperContig supercont3.388: 43,154-43,850](https://www.vectorbase.org/Culex_quinquefasciatus/Location/View?db=core;g=CPIJ012472;r=supercont3.388:43154-43850;t=CPIJ012472-RA) |
| CPIJ012471 | protease m1 zinc metalloprotease | [SuperContig supercont3.388: 2,033-6,778](https://www.vectorbase.org/Culex_quinquefasciatus/Location/View?db=core;g=CPIJ012471;r=supercont3.388:2033-6778;t=CPIJ012471-RA) |
