# Supplementary figures and images for: Identification of protease m1 zinc metalloprotease conferring resistance to deltamethrin by characterization of an AFLP marker in Culex pipiens pallens
Source: Parasit Vectors. 2016 Mar 23;9:172. doi: 10.1186/s13071-016-1450-4 (PMC4806500; doi:10.1186/s13071-016-1450-4)

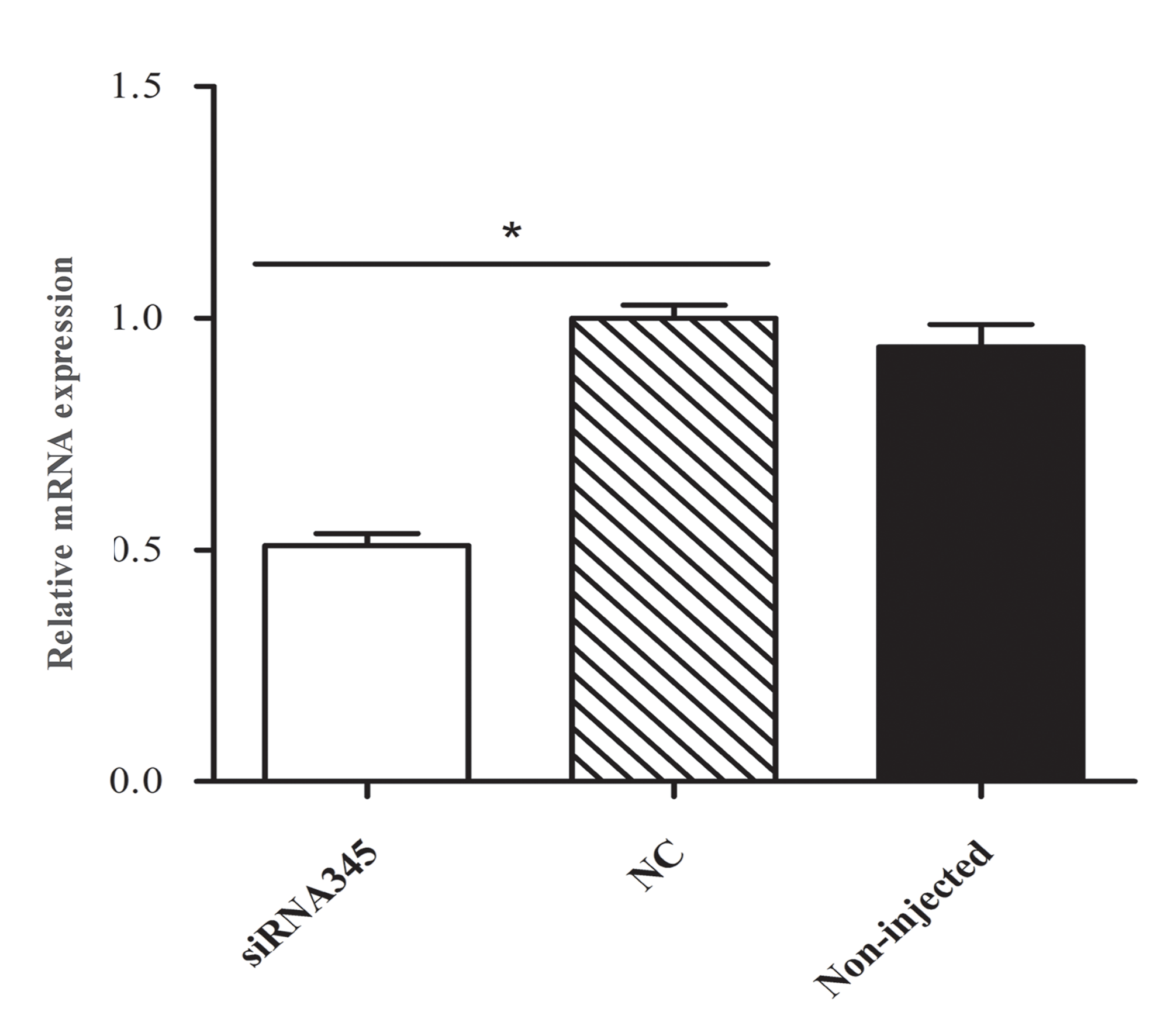

Supplement: Additional file 4: — RNAi efficiency verification of protease m1 zinc metalloprotease by qRT-PCR. The expression level of protease m1 zinc metalloprotease was down-regulated about 50 % in Lab-DS mosquitoes microinjected with the siRNA345, compared with that in mosquitoes injected NC RNA. The results were shown as the mean ± S.E. The significant difference was indicated by *(P < 0.05). (TIF 3435 kb) [file 13071_2016_1450_MOESM4_ESM.tif]

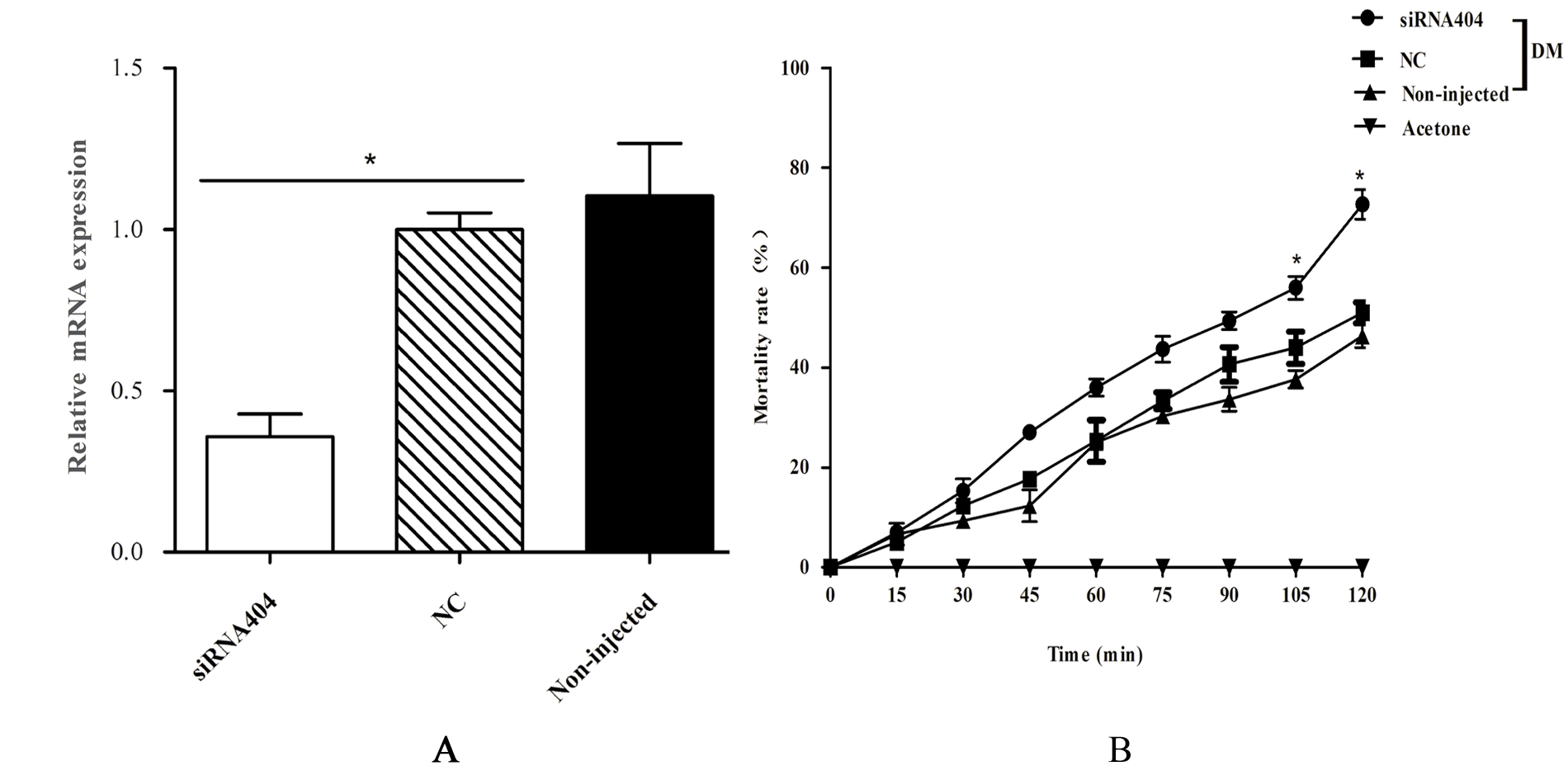

Supplement: Additional file 6: — (A) RNAi efficiency verification of CYP6CP1 by qRT-PCR. The expression level of CYP6CP1 gene was down-regulated about 65 % in Lab-DR4 mosquitoes microinjected with the siRNA404, compared with that in mosquitoes injected NC RNA. (B) Functional study of CYP6CP1 in Lab-DR4 strain. Mortalities of microinjected mosquitoes were observed after a 2 h exposure to CDC bottles treated with deltamethrin (4 mg/ml). The siRNA404 microinjected group had a higher mortality rate than the negative control. (TIF 1066 kb) [file 13071_2016_1450_MOESM6_ESM.tif]
