# Supplementary material for: Identification of protease m1 zinc metalloprotease conferring resistance to deltamethrin by characterization of an AFLP marker in Culex pipiens pallens
Source: Parasit Vectors. 2016 Mar 23;9:172. doi: 10.1186/s13071-016-1450-4 (PMC4806500; doi:10.1186/s13071-016-1450-4)
Supplement: Additional file 5: — The ORF sequences of protease m1 zinc metalloprotease in Lab-DS and Lab-DR4 strains. (DOCX 12 kb) [file 13071_2016_1450_MOESM5_ESM.docx]

**Additional File 5**

**The ORF sequence of *protease m1 zinc metalloprotease* in the Lab-DS strain of *culex pipiens pallens***

ATGAACAACAGCAACAACGCCAAGATGCAAATTCGGGAGAATTTCTCCGTGGACGGAACGGTGCGGTCCTCGTACGGCAGTGAACCACGTGGGTTCTTCATCACGCGGATGGCCTTGCTTACAACGGTTGCGATTTTCAGCTGTTTGCTGATTGGGACGGGACTGCTGATCTACCATCTGGCCGGATGTCCGGACGAGTCCGGTCCGGTTCACGCCAGCATTTGTGACCATCACCACTTTCATCACACGTTTGAGGCTTCGAATCTGAGTCGATTGGTGCCACATGACACGACCAGTACGGAGCCGGTTACGACCTCTACCAGTACTGCGAGCTCATCAACAACGGCCAAACTGGACAGTCAGGCTTCAGGAAATGGCTCTGACTCTCAAACTACTACTCAGCTGGAAGACGACGCCGATGTCCGGTTGCCACGGTCCGTCGAGCCGCTGTCCTACAACATCCGACTAATCCCATTCATGTTCGGGGACAACTTTACCTTTACCGGAACCGTTGACATTGAGGTACGCGTGCTCGAAGACTGCGACAACATAACACTCCACGCGGTCGCCCTGAAAATCCACGAAGCCCGCATGGAGCAGCAAAGACCACCACCAAAGACACGTGCGACACGTGCAACAGGTGCCGGTCTGAATGACAGCTACGAGTACGACGATGACGGTGATGATGACAATGATGTTGGTGGAGGAAATCGAACCGTCGTTGAAATTGAGCAACAGGTAGTCGTGGAGAGCAAACAGTTCTACGTGCTCAAGATGAGCCGGAAGCTGCGGGTCGGGGAGCGGTACACGGTGCGCATTCGGTACGAGGGAGTGCTGAACGACTATCTGCAAGGATTCTATCGCAGCTCGTACACCGTGAGGAATGAGACGAGTCAGGCAACTATGCCAGAAAAGTACCCGTAG

**The ORF sequence of *protease m1 zinc metalloprotease* in the Lab-DR4 strain of *culex pipiens pallens***

ATGAACAACAGCAACAACGCCAAGATGCAAATTCGGGAGAATTTCTCCGTGGACGGAACGGTGCGGTCCTCGTACGGCAGTGAACCACGTGGGTTCTTCATCACGCGGATGGCCTTGCTTACAACGGTTGCGATTTTCAGCTGTTTGCTGATTGGGACGGGACTGCTGATCTACCATCTGG

CCGGATGTCCGGACGAGTCCGGTCCGGTTCACGCCAGCATTTGTGACCATCACCACTTTCATCACACGTTTGAGGCTTCGAATCTGAGTCGATTGGTGCCACATGACACGACCAGTACGGAGCCGGTTACGACCTCTACCAGTACTGCGAGCTCATCAACAACGGCCAAACTGGACAGTCAGGCTTCAGGAAATGGCTCTGACTCTCAAACTACTACTCAGCTGGAAGACGACGCCGATGTCCGGTTGCCACGGTCCGTCGAGCCGCTGTCCTACAACATCCGACTAATCCCATTCATGTTCGGGGACAACTTTACCTTTACCGGAACCGTTGACATTGAGGTACGCGTGCTCGAAGACTGCGACAACATAACACTCCACGCGGTCGCCCTGAAAATCCACGAAGCCCGCATGGAGCAGCAAAGACCACCACCAAAGACACGTGCGACACGTGCAACAGGTGCCGGTCTGAATGACAGCTACGAGTACGACGATGACGGTGATGATGACAATGATGTTGGTGGAGGAAATCGAACCGTCGTTGAAATTGAGCAACAGGTAGTCGTGGAGAGCAAACAGTTCTACGTGCTCAAGATGAGCCGGAAGCTGCGGGTCGGGGAGCGGTATACGGTGCGCATTCGGTACGAGGGAGTGCTGAACGACTATCTGCAAGGATTCTATCGCAGCTCGTACACCGTGAGGAATGAGACGAGTCAGGCAACTATGCCAGAAAAGTACCCGTAG
